# Assessment of the Immunomodulatory Properties of the Probiotic Strain *Lactobacillus paracasei* K5 In Vitro and In Vivo

**DOI:** 10.3390/microorganisms8050709

**Published:** 2020-05-11

**Authors:** Pelagia Chondrou, Athanasios Karapetsas, Despoina Eugenia Kiousi, Stavros Vasileiadis, Petros Ypsilantis, Sotiris Botaitis, Athanasios Alexopoulos, Stavros Plessas, Eugenia Bezirtzoglou, Alex Galanis

**Affiliations:** 1 Department of Molecular Biology and Genetics, Faculty of Health Sciences, Democritus University of Thrace, Alexandroupolis 68100, Greece; pelagiaho@gmail.com (P.C.); a.karapetsas@dundee.ac.uk (A.K.); dkiousi@mbg.duth.gr (D.E.K.); 2 Laboratory of Experimental Surgery and Surgical Research, Medical School, Faculty of Health Sciences, Democritus University of Thrace, Alexandroupolis 68100, Greece; vasiliadis_stavros@hotmail.com (S.V.); pipsil@med.duth.gr (P.Y.); smpotait@med.duth.gr (S.B.); 3 Laboratory of Microbiology, Biotechnology and Hygiene, Faculty of Agricultural Development, Democritus University of Thrace, Orestiada 68200, Greece; alexopo@agro.duth.gr (A.A.); splessas@agro.duth.gr (S.P.); 4 Laboratory of Hygiene and Environmental Protection, Medical School, Faculty of Health Sciences, Democritus University of Thrace, Alexandroupolis 68100, Greece; empezirt@med.duth.gr

**Keywords:** probiotics, cytokines, air pouch, immunomodulation, Toll-like receptors

## Abstract

*Lactobacillus paracasei* K5 is a lactic acid bacteria (LAB) strain that has been isolated from dairy products. Previous studies have established its probiotic potential in a series of in vitro tests, including molecular characterization, safety profiling, and tolerability of the gastrointestinal tract conditions. To characterize its beneficial actions on the host, we have shown previously that *L. paracasei* K5 adheres to Caco-2 cells and exerts anti-proliferative effects through the induction of apoptosis. In the present study, we focused on the immunomodulatory potential of this strain. We employed the dorsal-air-pouch mouse model of inflammation and recorded an eight-fold increase in the recruitment of immune cells in mice treated with the probiotic strain, compared to the control group. Analysis of the exudates revealed significant changes in the expression of pro-inflammatory mediators on site. Treatment of Caco-2 cells with *L. paracasei* K5 induced significant upregulation of cytokines interleukin-1α (IL-1α), ΙL-1β, IL-6, tumor necrosis factor-alpha (TNF-α), the chemokine C-X-C motif ligand 2 (CXCL2), and the inflammation markers soluble intercellular adhesion molecule (sICAM) and metallopeptidase inhibitor-1 (TIMP-1). Transient induction of the Toll-like receptors (TLRs) 2, 4, 6, and 9 expression levels was recorded by real-time PCR analysis. These results highlight the immunomodulatory potential of this strain and further support its probiotic character.

## 1. Introduction

Probiotics are defined as viable microorganisms that, when administered in adequate quantities, confer a health benefit on the host [1]. Numerous studies have provided compelling evidence for the health promoting effects of probiotics on the host, including alleviation of the symptoms of irritable bowel syndrome [2]; relief of *Clostridium difficile*–associated diarrhea [3]; management of allergic disorders, such as atopic dermatitis (eczema) and allergic rhinitis [4]; and reduction of serum cholesterol [5]. Several Lactobacillus species have been employed as probiotics, including *Lactobacillus casei*, *L. plantarum*, and *L. rhamnosus*. Still, the probiotic properties typically vary and differ among the various strains; thus, the identification and in-depth characterization of probiotic strains is of high importance prior to their utilization in the food industry.

The health-promoting effects of the probiotic species are exerted through several cellular and molecular mechanisms, such as adhesion to the intestinal epithelium [6], competition with invading pathogens [7], reinforcement of the epithelial barrier, and production of antimicrobial molecules [8]. Moreover, a plethora of in vitro and in vivo studies has shown that probiotic bacteria can stimulate different cell populations of the immune system and modulate the responses [9]. For instance, probiotics can interact with the intestinal mucosa and residing immune cells, such as lymphocytes, macrophages, or dendritic cells, and trigger the production of cytokines, chemokines, or immunoglobins [10]. The immunomodulatory activities of probiotic bacteria are mainly mediated through the activation of TLRs. TLRs are expressed in a plethora of cells, including the intestinal epithelial cells (IEC) that line the gastrointestinal tract [11]. Post-stimulation TLRs trigger cellular cascades, leading to the activation of key signaling molecules, such as Nuclear Factor-kappa B (NF-κΒ) and p38 Mitogen Activated Protein Kinase (MAPK), that orchestrate inflammatory and adaptive immunity responses [11]. 

*Lactobacillus paracasei* K5 is a lactic acid bacteria (LAB) strain that has been isolated from dairy products [12]. Its identification and molecular characterization were performed by Multiplex PCR and Random Amplification of Polymorphic DNA (RAPD) analysis, while its probiotic potential was established through a series of in vitro tests that simulate the acidic conditions of the stomach and evaluate its safety for consumption [12]. Furthermore, its utilization as a starter culture in the production of novel functional foods and drinks, such as soft white cheese, pomegranate juice, and sourdough bread has been reported before [13,14,15]. Additionally, we demonstrated recently that *L. paracasei* K5 was able to adhere efficiently to Caco-2 colon cancer cells and inhibit their proliferation in a time- and dose-dependent manner. The observed antiproliferative effect was elicited mainly through induction of apoptosis by altering the expression of specific apoptosis-related genes [16].

Here, we investigated the immunomodulatory properties of *L. paracasei* K5, to further explore and validate its probiotic character. By employing the dorsal-air-pouch model of inflammation, we studied the recruitment of immune cells in mice treated with the probiotic strain. The expression levels of a range of cytokines and chemokines were also examined in the air-pouch exudates, as well as in vitro in epithelial colon cancer cells. Finally, the expression of specific TLRs was also studied in time-course experiments, and a preliminary investigation of the downstream signaling pathway involved was performed.

## 2. Materials and Methods 

### 2.1. Cell Culture

Human colorectal adenocarcinoma cell line Caco-2 was purchased from the American Type Culture Collection (ATCC). Cells were cultured in Dulbecco’s Modified Eagle Medium: Nutrient Mixture F12 (DMEM-F12) with L-alanyl L-glutamine stable glutamine and was enriched with 10% fetal bovine serum (FBS), 100 U/mL penicillin, and 100 μg/mL streptomycin (all from Biosera, Boussens, France). Caco-2 cultures were maintained at 37 °C, 5% CO_2_, in a humidified atmosphere, under sterile conditions. Cells were seeded in plastic 100 mm plates, at a density of 2 × 10^6^ cells.

### 2.2. Bacterial Strains and Culture Conditions

*L. paracasei* K5 was isolated from feta-type cheese [12]; *L. casei* ATCC 393 and *L. rhamnosus* GG ATCC 53103 were obtained from the German Collection of Microorganisms and Cell Cultures GmbH (DSMZ) (Braunschweig, Germany). All LAB strains were cultured in De Man, Rogosa, and Sharpe (MRS) broth (Merck, Darmstadt, Germany), in anaerobic conditions, at 37 °C, for 24 h. The bacterial cells were harvested by centrifugation (2.600 *g*/15 min) at stationary phase, washed twice with sterile phosphate-buffered saline (PBS) buffer (Biosera, Boussens, France), and adjusted to the appropriate density in DMEM-F12 medium containing 10% FBS 20 mM 4-(2-hydroxyethyl)-1-piperazineethanesulfonic acid (HEPES) (all from Biosera, Boussens, France), for the in vitro experiments, or in sterile PBS for the in vivo experiments.

### 2.3. Animals 

Six- to 8-week-old ΒALB/c male mice, weighing about 20–25 g, were bred in the Animal House unit of the Laboratory of Experimental Surgery and Surgical Research, Department of Medicine, Democritus University of Thrace. They were housed in polycarbonate cages, under controlled environmental conditions (20–23 °C, 12 h photoperiod), and had free access to tap water and standard commercial pelleted diet (certified rat chow, VIOZOIS S.A., Thessaloniki, Greece). The experimental protocol, as described below, in Section 2.4, was approved by the Animal Care and Use Committee of our Institution (license number 1089/02-02-2016), in accordance with the requirements set by PD 56/2013, which complied with Directive 2010/63/EU regarding protection of animals used for scientific purposes. Each group included in the present study consisted of five animals, and each experiment was conducted twice. Mice were euthanized after the experiment, by cervical dislocation.

### 2.4. Air-Pouch Formation and Exudate Collection

**Figure 1:**
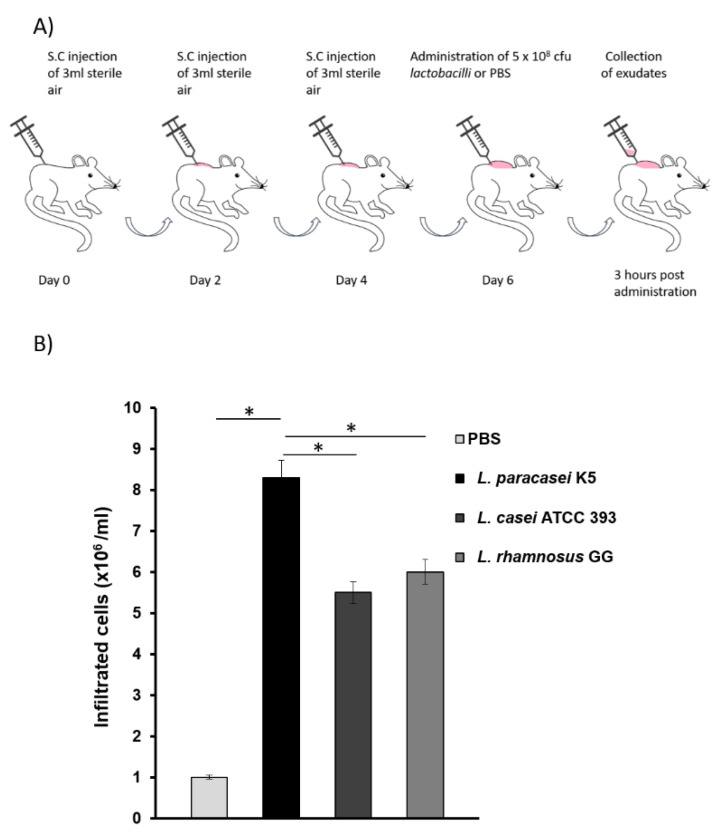
Evaluation of the immunomodulatory effect of *Lactobacillus paracasei* K5, using the mouse air-pouch model. *L. casei* ATCC 393 and *L. rhamnosus* GG were also tested as reference strains. (**A**) Schematic representation of the mouse dorsal-air-pouch model. Control animals received only PBS. (**B**) Total number of the infiltrated leukocytes in the exudates of air pouches. *L. paracasei* K5, as well as probiotic strains *L. casei* ATCC 393 and *L. rhamnosus* GG, was injected in the air pouches raised in wild-type BALB/c mice. The data shown are the mean of 5 mice per group ± standard deviation. * Significantly different from animals that received *L. paracasei* K5 (*p* < 0.05).

Air pouches were formed on the back of mice, as previously described [17] by subcutaneous injection of 3 mL sterile air (day 0), followed by injections in a day-by-day basis (days 2 and 4), for the formation of an equal size air pouch in each mouse (Figure 1A). On day 6, 5×10^8^ cfu of *L. paracasei* K5, *L. casei* ATCC 393, or *L. rhamnosus* GG resuspended in 200 μL sterile PBS was injected in dorsal air pouches, while 200 μL of sterile PBS was injected in the air pouch of control animals. Three hours post-injection, the exudates were collected by injecting 2 mL sterile PBS, followed by air-pouch lavage. The samples were centrifuged at 2.600× *g* for 15 min. The supernatants were collected, filtered through 0.22 μm filters, and used for the detection of cytokines and chemokines, while the cell pellets were examined for the presence of infiltrated leukocytes. The leukocyte populations were assessed by using a hemocytometer and trypan blue exclusion assay.

### 2.5. Detection of Cytokines and Chemokines in the Air-Pouch Exudates 

For the detection of cytokines and chemokines in the air-pouch exudates, the Proteome Profiler Mouse Cytokine Array kit (R&D Systems Inc., Minneapolis, MN, USA) was used by following the manufacturer’s protocol. Briefly, the filtered supernatants were mixed with a cocktail of 40 different biotinylated detection antibodies. The mixture was then incubated with the Mouse Cytokine Array membrane, overnight, at 4 °C. Following washing to remove unbound material, the membranes were incubated with Streptavidin-HRP (R&D Systems Inc., Minneapolis, MN, USA) for 30 min, at room temperature, and washed again, followed by chemiluminescence detection. 

### 2.6. Treatment of Caco-2 Cells with Probiotic Bacteria 

Caco-2 cells (2×10^6^ cells/plate) were co-cultured with *L. paracasei* K5 (10^8^ cfu) or *L. rhamnosus* GG (10^8^ cfu) for the indicated timepoints (0, 2, 4, 8, 12, and 16 h), in DMEM-F12 medium containing 10% FBS and 20 mM HEPES, and were maintained at 37 °C, 5% CO_2_, in a humidified atmosphere, under sterile conditions. After the incubation period, the bacteria were removed by aspiration, the cells were washed twice with PBS, and then they were collected for RNA extraction. Control cells were maintained at the same conditions in DMEM-F12 medium containing 10% FBS and 20 mM HEPES.

### 2.7. RNA Extraction and cDNA Synthesis

Total RNA from Caco-2 cells was extracted, using RNAiso Plus (Takara, Saint-Germain-en-Laye, France), following manufacturer’s instructions. The concentration and purity of the extracted RNA were evaluated spectrophotometrically and by agarose gel electrophoresis. Then, cDNA was synthesized from 4 μg total RNA, by using PrimeScript 1st Strand cDNA Synthesis Kit (Takara, Saint-Germain-en-Laye, France), following the manufacturer’s protocol.

### 2.8. Real Time PCR

Real-time PCR was performed using by the KAPA SYBR® FAST qPCR Kit (Kapa Biosystems, Wilmington, MA, USA), according to manufacturer’s instructions. Each reaction mixture consisted of a 20 μL total volume solution containing 10 μL SYBR Premix, 0.4 μL of forward and reverse primer, 2 μL cDNA, and 7.2 μL ddH_2_O. The reactions were performed on a StepOne PCR System in MicroAmp® Fast Optical 48-Well Reaction Plates (both from ThermoFisher Scientific, Loughborough, UK), at the following conditions: 95 °C for 3 min, followed by 40 cycles of 95 °C for 15 sec and 60 °C for 1 min. The housekeeping gene *b-actin* served as an endogenous control. Each reaction was performed in duplicates, and each experiment included two non-template controls. The sequences of the primers used in the PCR are shown in Supplementary Materials Table S1. Primer specificity was verified by performing a melting curve analysis. For the relative quantification of gene expression, the formula RQ=2^-ΔΔCt^ was used.

### 2.9. Statistical Analysis

GraphPad Prism (La Jolla, CA, USA) was used for statistical analysis of the data and construction of graphs. Results are represented as the mean ± standard deviation. Statistical differences were analyzed by 2-tailed Student’s *t*-tests. A *p*-value < 0.05 was considered statistically significant. Each in vitro experiment was replicated three independent times. For the in vivo experiments, each group consisted of five animals, and each experiment was conducted twice.

## 3. Results and Discussion

### 3.1. L. paracasei K5 Triggered the Infiltration of Leukocytes and the Expression of Cytokines and Chemokines in the Exudates of the Air Pouches

The immunomodulatory potential of *L. paracasei* K5 was initially investigated in vivo by employing the dorsal-air-pouch mouse model. The commercially available probiotic strains *L. casei* ATCC 393 and *L. rhamnosus* GG were utilized as reference. To this end, 5 × 10^8^ cfu of *L. paracasei* K5 were injected in the air pouches raised in wild-type BALB/c mice after six days (Figure 1A). As shown in Figure 1B, the injection of *L. paracasei* K5 induced a rapid and strong recruitment of leukocytes in the exudates of air pouches, significantly higher than *L. casei* ATCC 393 and *L. rhamnosus* GG (*p* < 0.05). Specifically, the administration of *L. paracasei* K5 increased the infiltration of leukocytes in the exudates by eight-fold compared to control mice that received only the PBS solution, whereas the administration of *L. casei* ATCC 393 and *L. rhamnosus* GG enhanced the recruitment of leukocytes up to six-fold compared to the control, as recorded before [18].

**Figure 2:**
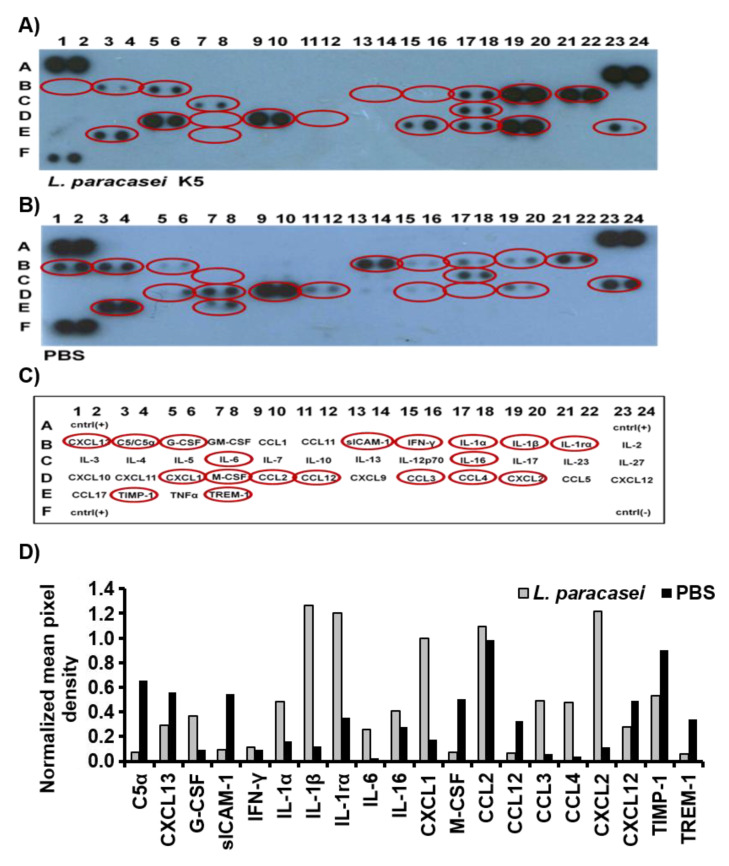
Effect of *L. paracasei* K5 on cytokine and chemokine production in the air pouch. *L. paracasei* K5 (**A**) or PBS (**B**) were administered in the air pouches of BALB/c mice, and after three hours, the exudates were collected, and the supernatants were analyzed for cytokine/chemokine expression, using the Proteome Profiler TM Mouse Antibody Array Panel A. The cytokines and chemokines with differential expression are encircled in red (**C**). The positions of the anti-cytokine and anti-chemokine antibodies (in duplicates) on the membranes are also indicated. The pairs of spots in the upper-left, upper-right, and lower-left corners are positive controls. (**D**) Data from (A,B) were quantified as mean pixel density normalized to the density of the positive controls. The use of two replicates per analyte limits the application of robust statistics.

To further characterize the immunomodulatory effects of *L. paracasei* K5, the exudates of dorsal air pouches were analyzed for the presence of cytokines and chemokines, by utilizing antibody arrays that permit the simultaneous detection and semi-quantitative analysis of 40 different cytokines/chemokines (Figure 2). The administration of *L. paracasei* K5 led to the differential expression of several cytokines and chemokines in the air-pouch exudates compared to mice that received only the PBS solution (Figure 2). In particular, *L. paracasei* K5 induced in the air-pouch exudates had higher levels of granulocyte-colony stimulating factor (G-CSF); interleukins IL-1α, IL-1β, IL-1ra, IL- 6, and IL-16; and chemokines CCL3, CCL4, CXCL1, and CXCL2, compared to the exudates from control mice that received only PBS. Moreover, the administration of *L*. *paracasei* K5 caused decreased expression levels of sICAM, TIMP-1, complement component 5α (C5α), macrophage colony-stimulating factor (M-CSF), and triggering receptor expressed on myeloid cells 1 (TREM-1) (Figure 2D). The cytokines and chemokines with differential expression are shown encircled in Figure 2C. The results of this semi-quantitative analysis are summarized in Table 1. 

Distinct modulation of the immune response in vivo following probiotic administration has been well documented. For example, we have showed previously that administration of *L. pentosus* B281 and *L. plantarum* B282, two recently characterized probiotic strains, induced significant recruitment of leukocytes in the air-pouch exudates of mice and differential expression of specific cytokines and chemokines [18]. Similarly, the probiotic strain *Lactobacillus casei* BL23 promoted a Th17-biased immune response, followed by the expression of proinflammatory cytokines (IL-6, IL-17, IL-10, and TGF-β), in a mouse model of intestinal inflammation [19]. Moreover, it has recently been demonstrated that oral treatment of BALB/c mice with the probiotic strain *L. fermentum* UCO-979C caused a significant increase in the production of interferon-γ (IFN-γ) and IL-6, as well as the stimulation of intestinal and peritoneal macrophages and the maturation of B cells [20]. 

Importantly, the infiltration of immune cells promoted by probiotic administration has been linked to beneficial health effects to the host. Indeed, it has been showed recently that the preemptive per os administration of *L. casei* ATCC 393 to colon CT26 syngeneic tumor mice model led to changes in the tumor microenvironment, which favored Th1 immunological responses and the recruitment of CD3^+^CD8^+^ cytotoxic T cells. More specifically, the production of granzyme B, TNF-α, and IL-12 was enhanced, promoting the maturation and migration of CD3^+^CD8^+^ cytotoxic T cells, leading to an almost four-fold increase of their population, in situ. These changes were correlated with a significant reduction in tumor volume, compared to control [21]. Likewise, Hu et al. used the same experimental model and showed that the per os administration of *L. plantarum* for 14 days enhanced CD8^+^ T and natural killer (NK) cells recruitment, leading to survival prolongation of the tumor-bearing mice [22]. Importantly, probiotics that modulate the recruitment of immunological populations to the tumor site could be used in combination with other immunotherapy drugs. Such an approach could aid clinicians in the treatment of chemo-resistant cancers. In this context, administration of the probiotic strain *Escherichia coli* strain Nissle 1917 to 4T1- and H22-subcutaneous-tumor-bearing BALB/c mice caused increased infiltration of CD3^+^CD8^+^ cytotoxic T cells. However, co-administration with galunisertib, a tumor growth factor beta (TGF-β) blockade agent, induced a more pronounced effect, as it enhanced the recruitment of dendritic cells and antigen-specific IFN-γ+CD8+ T cells within the tumor and in tumor-draining lymph nodes [23]. Clearly, this niche of probiotic research is very promising, but more studies should be conducted before the introduction of such practices in the clinic.

### 3.2. Induction of Cytokine and Chemokine Expression in Caco-2 cells upon Treatment with L. paracasei K5

**Figure 3:**
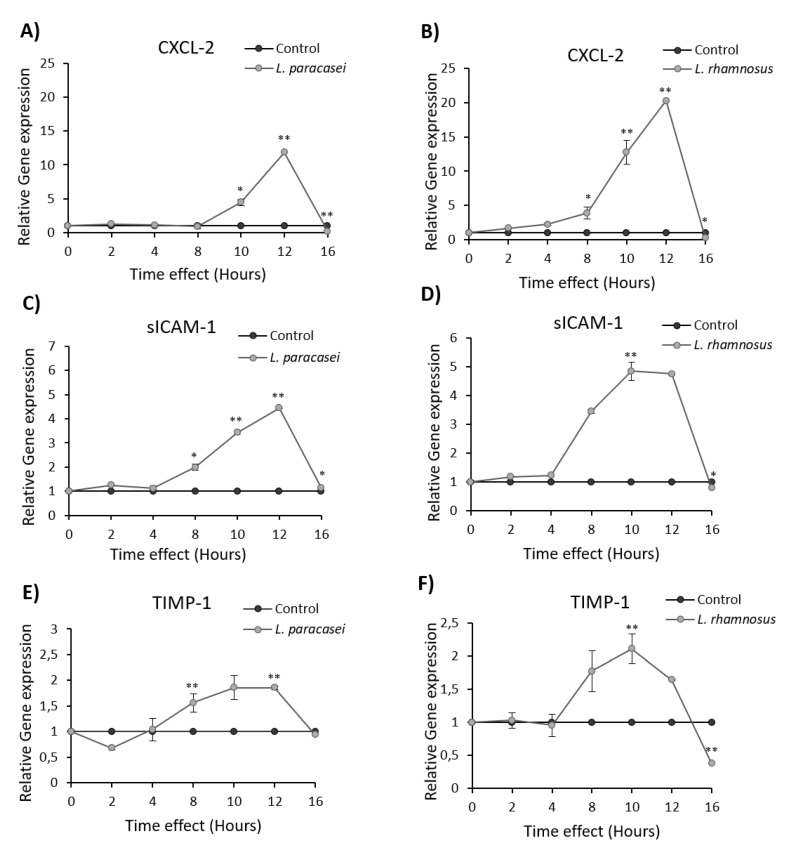
Expression levels of CXCL-2, sICAM-1, and TIMP-1 mRNA in Caco-2 cancer cell line induced by *Lactobacillus paracasei* K5 and *L. rhamnosus* GG. Cells were stimulated with *L. paracasei* K5 (**A**,**C**,**E**) or *L. rhamnosus* GG (**B**,**D**,**F**) in different time points (0, 2, 4, 8, 10, 12, and 16 h), and the relative expression of these genes was determined by qPCR. Control cells were treated only with DMEM F-12. The data presented are the mean ± standard deviation of three independent experiments. * *p* < 0.05; ** *p* < 0.01 compared to control.

**Figure 4:**
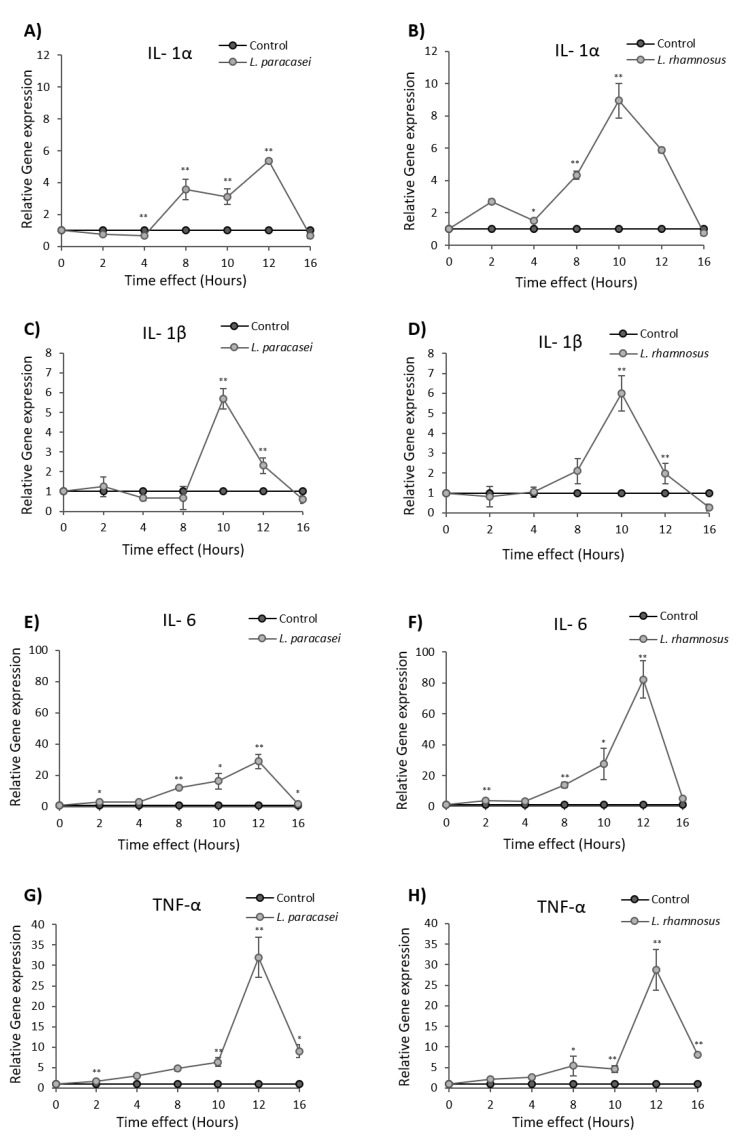
Expression levels of IL-1α, IL-1β, IL-6, and TNF-α mRNA in Caco-2 cancer cell line after *L. paracasei* K5 and *L. rhamnosus* GG treatments. Cells were stimulated with *L. paracasei* K5 (**A**,**C**,**E**,**G**) or *L. rhamnosus* GG (**B**,**D**,**F**,**H**) in different time points (0, 2, 4, 8, 10, 12, and 16 h), and the relative expression of these genes was determined by qPCR. Control cells were treated only with DMEM F-12. The data presented are the mean ± standard deviation of three independent experiments. * *p* < 0.05; ** *p* < 0.01 compared to control.

Although the mouse air-pouch system has been well established for studying the immunostimulatory effects of probiotic bacteria, it has been reported that the immune responses may vary in the air pouch and the gut intestine for certain probiotic strains [24]. In this vein, we studied the impact of the *L. paracasei* K5 on human colon cancer cells Caco-2, an in vitro system that simulates the intestinal epithelium. More specifically, the expression levels of the chemokine CXCL2, of the inflammation markers sICAM and TIMP-1 (Figure 3), as well as of the cytokines IL-1α, IL-1β, IL-6 and TNF-α (Figure 4), were investigated by qPCR upon treatment of Caco-2 cells with *L. paracasei* K5 in time-course experiments. The results showed that *L. paracasei* K5 treatments induced a time-dependent upregulation of the expression of CXCL-2, sICAM-1, and TIMP-1 (Figure 3A,C,E). All three markers peaked 12 h posttreatment, in a statistically significant manner, and returned to basal levels at 16 h. Treatment with the reference probiotic strain *L. rhamnosus* GG induced similar effects on the expression levels of the above genes. In particular, the maximum expression levels of CXCL-2 were observed 12 h posttreatment (Figure 3B) and of sICAM-1 and TIMP-1 were observed 10 h posttreatment (Figure 3D,F). After the 16-h mark, the expression levels of CXCL-2 and sICAM-1 returned to the basal state, while the levels of TIMP-1 reached a lower value (*p* < 0.01) (Figure 3F). Interestingly, the levels of sICAM and TIMP-1 were decreased in the air-pouch exudates, following *L. paracasei* K5 administration (Figure 2D). This difference could be attributed to the fact that a heterogenous cell population constitutes the air pouch and each cell type might respond differently to the probiotic strain rather than the uniform Caco-2 monolayer. The pro-inflammatory mediators IL-1α, IL-1β, IL-6, and TNF-α also showed a time-dependent increase in their relative expression levels that was counteracted after 16 h of treatments with either *L. paracasei* K5 or *L. rhamnosus* GG (Figure 4). More specifically, the expression of IL-1α, IL-6, and TNF-α peaked at 12 h posttreatment, while the expression of IL-1β reached its highest levels after 10 h of treatment. Of note, while the expression levels of IL-1α, IL-1β, and IL-6 returned to basal levels after 16 h, the increased expression of TNF-α persisted (Figure 4G,H).

These findings demonstrated that *L. paracasei* K5 induced an innate immune response through the time-regulated overexpression of specific pro-inflammatory cytokines and chemokines. In this line, it was demonstrated that treatment of intestinal epithelial cells with *L. acidophilus* NCFM enhanced the expression levels of the pro-inflammatory cytokines IL-1α and IL-1β and the chemokines CCL2 and CCL20, in a time-dependent manner [25]. Similarly, a transient overexpression of IL-1β, IL-6, and TNF-α was recorded in the Caco-2 cells stimulated by the probiotic strain *Lactobacillus plantarum* NDC 75017 [26]. In the same context, Caco-2 cells treated with *L. gasseri* CMUL34, *L. acidophilus* CMUL67, *L. gasseri* CMUL80, *L. gasseri* CMUL99, or *L. plantarum* CMUL140 showed a significant upregulation of IL-6 [27].

It is evident that probiotic bacteria can regulate host immune responses through the induction of a range of mediators from intestinal epithelial cells. However, supplementation with probiotics that induce pro-inflammatory effects could exacerbate immune responses in patients with inflammation-related disorders, such as inflammatory bowel disease (IBD), ultimately leading to unfavorable outcomes [28]. Therefore, characterization of the bioactive molecules of probiotics and elucidation of their mechanisms of action should be performed to confirm their beneficial effects on the host [29].

### 3.3. L. paracasei K5 Induced the Transient Expression of TLRs in Caco-2 Cells

**Figure 5:**
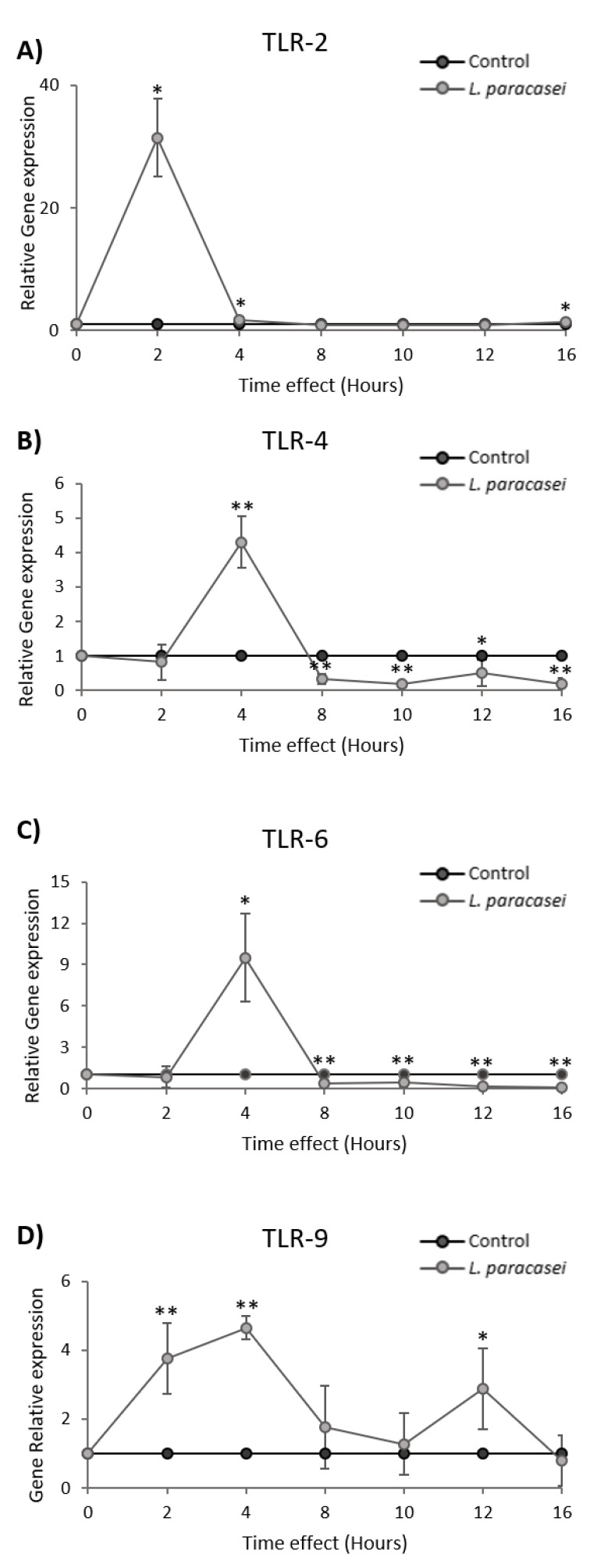
Expression levels of (**A**) TLR-2, (**B**) TLR-4, (**C**) TLR-6, and (**D**) TLR-9 mRNA in the Caco-2 colon adenocarcinoma cell line upon *L. paracasei* K5 stimulation in different time points (0, 2, 4, 8, 10, 12, and 16 h), as determined by qPCR. Control cells were treated only with DMEM F-12. The data presented are the mean ± standard deviation of three independent experiments. * *p* < 0.05; ** *p* < 0.01 compared to control.

Probiotic bacteria could evoke immune responses by interacting with extracellular and intracellular TLRs. In this vein, Caco-2 cells were treated with 10^8^ cfu of *L. paracasei* K5 in time-course experiments, and the expression levels of TLR-2, TLR-4, TLR-6, and TLR-9 were analyzed at the transcriptional level, using qPCR. The data showed a rapid but transient upregulation of the genes coding for TLRs following *L. paracasei* K5 treatments. More specifically, TLR-2 was strongly upregulated at two hours posttreatment, and then sharply declined to basal levels (Figure 5A). The maximum expression levels of TLR-4 and TLR-6 were observed at four hours posttreatment; however, they decreased to sub-basal levels at 12 h (*p* < 0.05) (Figure 5B,C). Of note, the expression pattern of TLR-9 after the stimulations was characterized by two peaks at four hours (*p* < 0.01) and at 12 h (*p* < 0.05). The expression levels of this gene returned to the basal state by the 16-h mark (Figure 5D). 

TLRs represent a receptor family employed by the innate immune system to recognize microbe- or host-specific antigens, called pathogen-associated molecular patterns (PAMPs) or damage-associated molecular patterns (DAMPs), respectively. It is well documented that many probiotic strains can orchestrate immune responses by modulating the expression of TLRs. Indeed, the probiotic strains *L. acidophilus* NCFM and *L. plantarum* NDC 75017 significantly upregulated the expression of TLR-2 in Caco-2 cells by stimulating the NF-κΒ and p38 MAPK signaling pathways, as shown in two independent studies [25,26]. In a similar manner, *L. rhamnosus* GG and *L. plantarum* BFE 1685 significantly elevated the transcriptional levels of TLR-2, TLR-5, and TLR-9 in HT-29 colon cancer cells [30]. The expression of TLR-9 was also upregulated after treatments of Caco-2 cells with cell-free supernatants or isolated nucleic acids of *L. johnsonii* N6.2. Such events led to the production of type 1 interferons, mimicking a low-grade viral infection, with potential prophylactic effects [31]. In the same context, two-hour treatments of HT-29 cells with the probiotic strain *L. johnsonii* BFE 6128 stimulated the expression of TLR-2, TLR-5, TLR-7, and TLR-9 and the simultaneous activation of the transcription factors interferon regulatory factor 1 (IRF), nuclear factor of light polypeptide gene enhancer in B cells 2 (NFKB2), and V-fos FBJ murine osteosarcoma viral oncogene homologue (FOS) [32]. Finally, it has been well documented that LAB strains may induce distinct inflammatory responses via the interaction with TLR-2 and TLR-4. For example, it has been shown that the interaction of *L. delbrueckii* subsp. *delbrueckii* TUA4408L and its extracellular polysaccharide (EPS), with TLR-2 and TLR-4, modulated the inflammatory response of porcine intestinal epithelial (PIE) cells against enterotoxigenic *E. coli* [33]. Similarly, *L. plantarum* N14 and its exopolysaccharide attenuated the production of specific pro-inflammatory cytokines from PIE cells, in response to enterotoxigenic *Escherichia coli* challenge, via TLR-2 and TLR-4 [34]. 

Finally, to initially characterize the downstream signaling pathways involved in the observed immunostimulatory activity of *L. paracasei* K5, Caco-2 cells were pretreated for one hour, either with a p38 MAPK specific inhibitor, SB203580, or with a vehicle stimulated with *L. paracasei* K5, and then the expression levels of the pro-inflammatory cytokines IL-1α, IL-1β, IL-6, and TNF-α were monitored by qPCR. Inhibition of the p38 MAPK pathway by SB203580 resulted in a significant decrease in the expression of IL-1α, IL-1β, IL-6, and TNF-α, 12 h post *L. paracasei* K5 administration (Supplementary Figure S1). Notably, previous studies from our team have shown that *L. paracasei* K5 may have direct anti-proliferative effects on Caco-2 cells by inducing apoptosis [16]. Evidently, it would be of particular interest to explore further the signaling pathways associated with the molecular mechanisms of action of *L. paracasei* K5, to elucidate and establish its probiotic character.

## 4. Conclusions

In the present study, we investigated the immunomodulatory potential of *L. paracasei* K5, in vitro and in vivo, to further support its probiotic character. Delineating the immunomodulatory potential of probiotics is of great importance for the determination of their safety. Indeed, administration of probiotics that exhibit high immunostimulatory effects should potentially be avoided for consumers with chronic inflammation. In this context, the guidelines for the evaluation of probiotics in food issued by FAO/WHO clearly state that human trials should also be performed, to validate the safety and efficacy of probiotics prior to their application in the food industry [1].

Conclusively, our results showed that the injection of *L. paracasei* K5 to dorsal air pouches of BALB/c mice caused the enhanced recruitment of leukocytes on site (eight-fold), compared to mice that received sterile PBS. Analysis of the exudates revealed elevated levels of various pro-inflammatory factors and chemokines. The immunomodulatory effects of this strain were further confirmed in vitro, in time-course experiments. Indeed, treatment with *L. paracasei* K5 induced transient changes in the expression of the pro-inflammatory mediators IL-1α, ΙL-1β, IL-6, TNF-α, CXCL2, sICAM, and TIMP-1. Concomitantly, rapid upregulation of the expression of TLRs was demonstrated. Indeed, the levels of TLR-2, TLR-4, and TLR-6 were sharply elevated in less than four hours, while the levels of TLR-9 remained high throughout the time course, before reaching basal levels at 16 h. Lastly, preliminary experiments with a p38 MAPK inhibitor indicated a possible involvement of the p38 MAPK pathway in *L. paracasei* K5 immunostimulatory action. More studies are required to fully understand the cellular processes that take place upon stimulation with *L. paracasei* K5. 

## Figures and Tables

**Table 1:** Differential expression of cytokines, chemokines, and other inflammatory markers, as a result of air-pouch exposure to *L. paracasei* K5.

Markers	*L. paracasei* K5
IL-1α	↑ ^a^
IL-1β	↑
IL-1rα	↑
IL-16	↑
IL-6	↑
C5a	↓
CCL2	nd ^b^
CCL3	↑
CCL4	↑
CCL12	↓ ^c^
IFN-γ	nd
sICAM-1	↓
CXCL1	↑
CXCL2	↑
CXCL12	↓
CXCL13	↓
G-CSF	↑
M-CSF	↓
TIMP-1	↓
TREM-1	↓

^a^ Upregulation; ^b^ no detection; ^c^ downregulation.

## Supplementary Materials

The following are available online at https://www.mdpi.com/2076-2607/8/5/709/s1. Figure S1: Percentage of the reduction of the relative expression of genes encoding IL-1α, IL-1β, IL-6, and TNF-α in Caco-2 cells pretreated with the inhibitor SB203580 (p38 inhibitor) or vehicle and then cultured with *L. paracasei* K5 for 12 h. Table S1: The primers used at the quantitative real-time PCR analysis.
