# Supplementary material for: Assessment of the Immunomodulatory Properties of the Probiotic Strain Lactobacillus paracasei K5 In Vitro and In Vivo
Source: Microorganisms. 2020 May 11;8(5):709. doi: 10.3390/microorganisms8050709 (PMC7284587; doi:10.3390/microorganisms8050709)
Supplement: Supplementary file 1 [file microorganisms-08-00709-s001.zip › Supplementary Figure S1.docx]

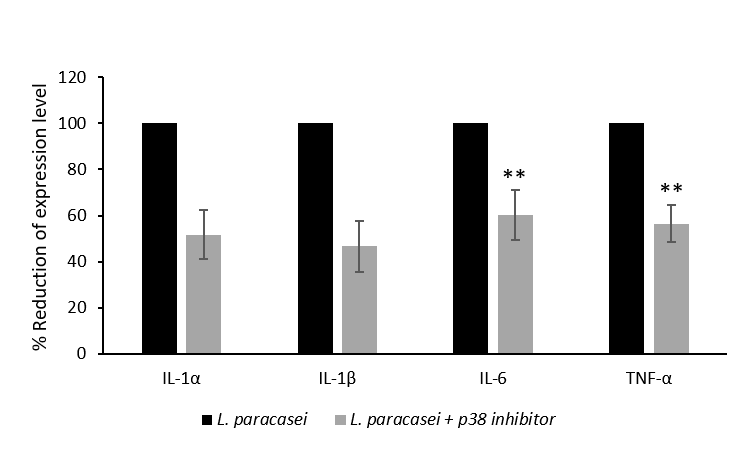


**Figure S1.** Percentage of the reduction of the relative expression of genes encoding IL-1α, IL-1β, IL-6 and TNF-α in Caco-2 cells pre-treated with the inhibitor SB203580 (p38 inhibitor) or vehicle and then cultured with *L. paracasei* K5 for 12 hours. Prior to stimulation with *L. paracasei* K5, Caco-2 cells were incubated with the p38 MAPK inhibitor (SB203580, 10μM; Sigma) for 1 hour. Then, the cells were washed twice with sterile PBS solution and exposed to *L. paracasei* K5 for 12 hours. The experiments were terminated by washing the cells with PBS and total RNA was extracted for conducting Real Time PCR. Gene expression was expressed as the mean ± standard deviation (SD) of three independent experiments * *p*<0.05; ** *p*<0.01 compared to negative control (untreated cells).
