# Supplementary material for: Assessment of the Immunomodulatory Properties of the Probiotic Strain Lactobacillus paracasei K5 In Vitro and In Vivo
Source: Microorganisms. 2020 May 11;8(5):709. doi: 10.3390/microorganisms8050709 (PMC7284587; doi:10.3390/microorganisms8050709)
Supplement: Supplementary file 1 [file microorganisms-08-00709-s001.zip › Supplementary Table S1.docx]

**Table S1.** The primers used at the quantitative real-time PCR analysis

| Gene | Forward primer sequence | Reverse primer sequence |
| --- | --- | --- |
| *TNF-α* | 5'-CCCAGGCAGTCAGATCATCTTC-3' | 5'-AGCTGCCCCTCAGCTTGA-3' |
| *β-ACTIN* | 5'-GCGCGGCTACAGCTTCA-3' | 5'-CTTAATGTCACGCACGATTTCC-3' |
| *TIMP-1* | 5'-GGTGGGTGGATGAGTAATGCA-3' | 5'-AGCGGGTGCGGAAACC-3' |
| *IL-1A* | 5'-TGTATGTGACTGCCCAAGATGAA-3' | 5'-TGGGTATCTCAGGCATCTCCTT-3' |
| *IL-1B* | 5'-CGAATCTCCGACCACCACTAC-3' | 5'-TCCATGGCCACAACAACTGA-3' |
| *IL-6* | 5'-AGGGCTCTTCGGCAAATGTA-3' | 5'-GAAGGAATGCCCATTAACAACAA-3' |
| *sICAM* | 5'-TCGAGATCTTGAGGGCACCTA-3' | 5'-CACAGTGATGATGACAATCTCATACC-3' |
| *CXCL-2* | 5'-TGTGATAGAGGCTGAGGAATCCA-3' | 5'-ACATTTCCCTGCCGTCACAT-3' |
| *TLR-2* | 5'-ATCCTCCAATCAGGCTTCTCT-3' | 5'-ACACCTCTGTAGGTCACTGTTG-3' |
| *TLR-4* | 5'-ATATTGACAGGAAACCCCATCCA-3' | 5'-AGAGAGATTGAGTAGGGGCATTT-3' |
| *TLR-6* | 5'-GGGACTCAGCATGGTAGAAGGTA-3' | 5'-CTCCTGTTACTCTGCAAGCTTTCA-3' |
| *TLR-9* | 5'-GGCAAAGTGGGCGAGATG-3' | 5'-GCTCTGCGTTTTGTCGAGAC-3' |
